# Neural mechanisms of sensory over-responsivity in youth with early caregiver-related adversity

**DOI:** 10.1186/s13034-026-01152-y

**Published:** 2026-08-08

**Authors:** Megan Banchik, Adriana S. Méndez Leal, Melis E. Cakar, Audra K. Langley, Jill M. Waterman, Nim Tottenham, Shulamite A. Green

**Affiliations:** 1 Department of Clinical Child Psychology, University of Kansas, Lawrence, KS USA; 2 University of Kansas Lifespan Institute, Lawrence, KS USA; 3 Department of Psychiatry and Biobehavioral Sciences, Jane & Terry Semel Institute for Neuroscience and Human Behavior, Los Angeles, CA USA; 4 Department of Psychology, University of California, Los Angeles, CA USA; 5 Neuroscience Interdepartmental Program, Ahmanson Lovelace Brain Mapping Center, University of California Los Angeles, Los Angeles, CA USA; 6 Los Angeles Pritzker Center for Strengthening Children and Families, University of California, Los Angeles, CA USA; 7 Department of Psychology, Columbia University, New York, NY USA

**Keywords:** Sensory over-responsivity, Early adversity, Sensory processing, FMRI

## Abstract

Sensory over-responsivity (SOR) is an impairing sensory processing challenge prevalent across a range of clinical and neurodevelopmental populations, as well as those with experiences of early caregiver-related adversity. Despite its prevalence and impairing nature, little research exists investigating SOR, and its distinct, underlying neural mechanisms, in early caregiver-related adversity. We examined SOR in children and adolescents ages 7–17 years old who were adopted from foster care (AFC; *n* = 31) and non-adopted comparisons (NAC; *n* = 26). Using functional magnetic resonance imaging, we investigated neural activity and functional connectivity in response to mildly aversive auditory and tactile stimulation and its relationship to SOR. The AFC group had elevated SOR scores as well as greater activation in somatosensory cortex compared to NAC. Within the AFC group, those with lower SOR showed increased activation of subcortical (thalamus, amygdala, hippocampus) and cortical (pre- and post-central gyri, and cingulate gyrus) regions. In the AFC group, negative functional-connectivity was observed between prefrontal and sensory regions, across sensory regions, and between sensory regulation and sensory reactivity-related regions. While sensory-evoked activation results suggest that children with early caregiver-related adversity show some hyperactivity to aversive sensory stimulation, consistent with other clinical groups, connectivity analyses suggest additional, unique mechanisms of SOR. Furthermore, functional-connectivity results provide evidence that children with early caregiver-related adversity may develop compensatory mechanisms that contribute to resilience for SOR. Results help explain individual differences in SOR in youth with early caregiver-related adversity and suggest a potential neural basis for some resilience to more severe SOR in these youth.

**Supplementary Information:**

The online version contains supplementary material available at https://doi.org/10.1186/s13034-026-01152-y.

## Introduction

Prenatal or early life environmental exposures that interfere with parent-child relationships (often described as early caregiving-related adversity) are both prevalent and varied, ranging from adverse living conditions (e.g., foster care or institutional care) to exposure to neglect, abuse, violence, parental mental illness or parent substance use disorders [54]. While these experiences differ in type, duration, and severity, experiences of early adversity often disrupt typical neural, cognitive, behavioral, and social-emotional development [7, 8, 25, 31, 48, 49, 57, 60]. This altered neurodevelopment may have downstream effects including increased susceptibility to stress-related internalizing and externalizing symptomatology [25, 26]. Furthermore, research suggests that children with significant early caregiver-related adversity such as removal from attachment figures, abuse, neglect, or multiple caregiving placements are at an increased risk for lower-level sensory processing challenges compared to their non-adopted peers, and that these sensory processing atypicalities may even contribute to or exacerbate emotional and behavioral challenges in this population [1, 25–27, 41].

Of the sensory processing challenges observed in youth with early adverse experiences, the best characterized is sensory over-responsivity (SOR), or an intensified negative reaction to aversive sensory stimuli. SOR can manifest in a variety of ways including avoidance of or discomfort with specific textures, bright lights, or loud noises, and therefore disrupt day-to-day life for those who struggle to modulate their responses to these stimuli. SOR has been associated with heightened anxiety levels and maladaptive behaviors as well as deficits in both social functioning and daily living skills, and may be correlated to worse outcomes in individuals with comorbid mental health disorders [11, 12, 36, 47].

Despite their apparent vulnerability to sensory processing challenges, the etiology of sensory challenges in youth with early caregiver-related adversity is still unclear. In typical development with stable early caregiving experiences, early attentional and affective responses are scaffolded by parents, guiding the interpretation of sensory signals and encouraging the development of top down regulatory pathways such as prefrontal regulation of subcortical arousal systems [14, 27, 35]. In contrast, unpredictable caregiving environments are associated with hyper-reactivity of the amygdala and disruptions to the development of prefrontal and thalamic regions that are responsible for sensory gating and emotion regulation [8, 15, 18, 29, 32, 50]. Neural risk may also precede postnatal caregiving disruption. Prenatal exposures to stress, substance use, or lack of access to prenatal care have been shown to independently alter similar sensory processing and emotion regulation networks including amygdala, thalamus, and prefrontal cortex [39, 44, 45, 58]. Thus, both prenatal and postnatal adversity, either independently or through compounding risk, may converge in a common neural profile, one which is primed for threat detection and less equipped to regulate the resulting arousal, and may struggle to process or modulate reactions to everyday sensory stimuli.

Although there are well-characterized impacts of early caregiving adversity on neurodevelopment, there are, as of now, no studies examining the functional neural correlates of SOR in this group. This study thus aims to identify the neurobiological mechanisms of SOR in individuals with early caregiver-related adversity, by comparing neural responses to aversive sensory stimuli in children and adolescents adopted from foster care (AFC) to their non-adopted peers as well as the relationship between these neural responses and SOR symptom severity.

Most research on the neurobiological mechanisms of SOR has been focused on children with Autism Spectrum Disorder, in part because atypical sensory reactivity is part of the diagnostic criteria for autism [2]. While there is little research on the neurobiology of SOR in early caregiving adversity, there is evidence of common neurological mechanisms across individuals with SOR and those with early caregiver-related adversity, which may help explain the prevalence of SOR in this group. Previous research on SOR in autism indicates that SOR is related to altered function of the amygdala, a neural structure thought to be the core of threat, emotion, and motivation processing, during sensory stimulation [18, 20], and exposure to early caregiver-related adversity has similarly been consistently shown to alter the typical course of amygdala development [10, 14, 33, 35, 50, 55].

There may also be unique neural mechanisms associated with SOR in early caregiver-related adversity exposed groups compared to other previously studied clinical populations. For example, previous research suggests that SOR in autism may be related to over-attribution of salience to extraneous sensory stimuli compared to social-emotional stimuli [18, 53] and SOR has been shown to contribute to atypical processing of social information in ASD (e.g., [19, 40, 53]. However, individuals with early adversity do not show the same level of social challenges with identification of social cues [23] despite relatively high levels of SOR, suggesting that SOR may not have the same direct impact on social attention, and suggest potentially different underlying or compensatory mechanisms of SOR in this group.

Given the need to understand neural mechanisms of sensory challenges in the early caregiver-related adversity population, this study aims to identify neural activation and connectivity during aversive sensory exposure in early caregiver-related adversity exposed individuals. Identifying the underlying neural mechanisms and unique characteristics of SOR in this group could then inform intervention development, specifically, the development of novel and targeted interventions tailored to the neural and behavioral presentation of SOR in early caregiver-related adversity exposed children and adolescents.

## Methods

### Participants

#### Recruitment

Participants included 58 children and adolescents aged 7.60–18.00 years (mean = 12.80; Table 1). Thirty-two were youth adopted from foster care (AFC) who had early caregiving-related adversity such as neglect, abuse, and multiple caregiving placements (see Supplemental Table S1). All AFC participants had finalized adoptions through the Los Angeles Department of Child and Family services (DCFS) after having been removed from their birth parents or voluntarily surrendered (mean age of removal from birth parents = 17.59 months; range = 0–72 months; mean age of final placement = 34.48 months; range = 0–108 months; Supplement Table S1). Non-adopted comparisons (NAC) were then pulled from a larger, previously published sample [20] of typically developing participants to create a comparison group of 26 NAC that matched the AFC group on age and sex. The two groups differed significantly in FSIQ (AFC mean = 99.31, range = 71–115; NAC mean = 110.65, range 78–133) as measured by the Wechsler Abbreviated Scales of Intelligence [56], so FSIQ was used as a covariate in all group comparisons. Exclusionary criteria included major mental illnesses (i.e., schizophrenia, bipolar disorder), seizure disorders, brain injury or lesions. To examine sensory processing in AFC as a distinct group separate from autism, all participants with a known or suspected diagnosis (i.e., parents are asked if there are any autism-related concerns either from themselves or from other providers such as teachers or therapists) of Autism Spectrum Disorder were excluded. The NAC group also could not have any history of developmental or cognitive delays or psychological disorders. Fifteen participants in the AFC group were taking psychoactive medication, and of these, thirteen were taking stimulant medication. Further details on medication are presented in the Supplement. None of the NAC group was taking psychoactive medication. Due to the large percentage of the AFC group taking stimulant medication, use of stimulant medication was included as a covariate in all between- and within- group analyses. Results comparing neural activation within the AFC group of those taking vs. not taking stimulants is presented in the Supplement.

**Table 1:** Demographics

	AFC	NAC	AFC vs. NAC	
	*N*		Chi squared	
Total	32	26		
Sex Assigned at Birth			1.55	
Female	20	14		
Male	12	12		
Handedness			0.001	
Left	3	4		
Right	27	22		
Race/Ethnicity			10.93	
Asian or Asian American, Hispanic or Latino/a	0	6		
Asian or Asian American, not Hispanic or Latino/a	0	1		
Black or African American	13	2		
Hispanic or Latino/a	1	2		
Multiracial, Hispanic or Latino/a	3	2		
Multiracial, not Hispanic or Latino/a	2	1		
White, Hispanic or Latino/a	4	3		
White, not Hispanic or Latino/a	9	9		

	**Mean (Std. Dev)**		**t-value**	
Age (years)	12.57 (2.89)	13.06(3.14)	−0.624	
Full Scale IQ	99.31(11.57)	110.65(12.71)	−3.55**	
Behavioral Measures				
SOR	3.90 (5.81)	1.08(1.55)	−2.59*	
SCARED	17.29 (15.42)	7.04 (6.13)	−3.40**	
Scanner Motion				
Mean Absolute Motion (mm)	0.52 (0.26)	0.40 (0.23)	1.647	
Mean Relative Motion (mm)	0.15 (0.05)	0.13 (0.05)	1.311	
Volumes Scrubbed	27.11 (16.07)	18.62 (15.61)	1.951	

SOR: sensory over responsivity; SCARED: Screen for Child Anxiety Related Disorders: AFC*:* children with early caregiver-related adversity adopted from foster care; NAC: non-adopted comparisons

* *p *< 0.05

**p < 0.01

***p < 0.001

#### Analysis groups

Behavioral analysis included 31 AFC (one participant did not have behavioral measures), and 26 NAC participants. Task-based fMRI analysis and analysis of functional connectivity included 26 AFC and 25 NAC (5 AFC and 1 NAC participants were excluded due to mean absolute motion in the scanner over 1.0 mm or mean relative motion greater than 0.2 mm). Bottom-up regression of the effect of SOR on neural activation included 25 AFC as one participant with usable imaging data did not have SOR scores.

### Behavioral measures

#### Sensory over-responsivity

The Sensory Over-Responsivity Inventory [37, 46, 52], a checklist of 52 potentially aversive sensory items, was completed for each participant by their parent. SOR was calculated by summing the number of items endorsed as causing an over-responsive reaction in auditory, visual, and tactile domains.

#### Anxiety

The Screen for Child Anxiety Related Disorders (SCARED) [5, 6] was completed for each participant by their parent to assess for anxiety and anxiety-related symptoms. As the SCARED has demonstrated inter-rater reliability, parent report was used over self-report to be consistent with the measure of SOR, and to be consistent across an age range that includes young children who may have trouble reporting on their own experiences [3]. The SCARED consists of 41 questions measuring anxiety symptoms across different categories.

### Sensory paradigm

During an 8.5-minute fMRI scan, participants experienced six 15-sec blocks each of alternating tactile, auditory, and joint (both tactile and auditory) conditions, with presentation order counterbalanced between subjects. This study focuses on the joint condition as it more closely resembles real-life multisensory exposure.

Throughout the scan, participants focused on a fixation cross, both while experiencing the sensory exposure as well as during 12.5-second rest periods between each block and at the beginning and end of the scan. The auditory stimulus was a pulsing violet noise (similar to white noise but with emphasis on lower frequencies). Tactile stimulation consisted of a scratchy bath glove rubbed on the inner left arm, applied from the wrist to the crease of the elbow by a tactile administrator. Stimuli were chosen via pilot testing in which neurotypical individuals rated each of multiple auditory and tactile stimuli on aversiveness from 0 to 10. Stimuli that were chosen were considered mildly aversive and rated an average of 4 out of 10 [20]. Tactile administrators underwent extensive reliability training, and pressure was tested on a research assistant prior to each administration to ensure pressure consistency across participants. Tactile administrators then followed a standardized timing protocol when administering tactile stimuli on each participant, alternating rubbing up then down the arm once per second. Prior to the task, participants were instructed to lie still, keep their eyes open, and look at a fixation cross (rest) in between each block.

### MRI acquisition

Scans were acquired on a Siemens Prisma 3-T MRI scanner. Each functional scan acquired 706 multiband echo planar imaging volumes (gradient-echo, TR=720ms, TE=37ms, flip angle = 52°, 104 × 90 matrix, 208 mm FOV, 72 slices, voxel size = 2 × 2 × 2 mm). The pre-scan normalize option was used to correct for signal inhomogeneities. To reduce interference from scanner noise, all participants wore earplugs, and the auditory stimulus was presented using noise-cancelling MRI-compatible headphones.

### fMRI analysis

#### Within- and between-group analysis of neural activation during sensory stimuli

Analyses were performed using the FMRIB Software Library (FSL) version 5.0.11. Preprocessing of single subject functional imaging included motion correction to the mean image, spatial smoothing (Gaussian kernel full width at half maximum=5 mm), and highpass temporal filtering (t > 0.01 Hz). Functional data was then linearly registered to a common stereotaxic space using the MNI152 T1 2 mm template (12 degrees of freedom). Analysis was performed using FSL’s fMRI Expert Analysis Tool (FEAT) version 6.00. Each experimental condition (auditory, tactile, and joint) was modeled with respect to rest. Fixed-effects models were run separately for each subject and included 12 motion parameters as covariates. The FSL Motion Outliers tool (refrms option) was also used to identify and regress out volumes that were outliers for motion. Because there were significant group differences on motion (Table 1), mean absolute motion was used as a covariate in between-group analyses. At the group level, subjects were combined in a higher-level mixed-effects model to investigate within-group activation and between-group differences. These higher-level group analyses were carried out using FSL’s Local Analysis of Mixed Effects State (FLAME 1 + 2). All higher-level within and between group analyses were corrected for multiple comparisons using Gaussian random-field theory in FSL (Z > 2.7) and cluster corrected (*p* <.05). All between-group contrasts were masked (post-hoc) by lower-thresholded (Z > 1.7 *p* <.05) within-group masks to determine whether group differences were driven by positive or negative activity (i.e., differences in activation or in deactivation).

#### Correlations of SOR symptomatology with neural activation

To investigate the relationship between neural activity during sensory stimulation and SOR symptoms in the AFC group, neural activity was correlated with SOR scores in a bottom-up regression (Z > 2.3 *p*<.05; Fig. 3). Due to the high co-occurrence of SOR and anxiety [16, 17], SCARED anxiety total scores were used as a covariate to identify neural activity that related to increased levels of SOR above and beyond the effects of anxiety (Fig. 3a). Analyses conducted without anxiety as a covariate are reported in the supplement (Figure S2). This analysis was performed only in the AFC group, as the NAC group had a very narrow range of SOR.

#### Psychophysiological Interaction

Psychophysiological interaction (PPI) analyses were used to examine patterns of functional connectivity during sensory exposure within the AFC group. PPI analyses examine the interaction between a task and time series of a given seed region to identify areas of the brain where change in activity is correlated with the seed region during the task compared to rest. These analyses were conducted to further explain the group differences and correlations with SOR seen in the prior two analyses. Thus, the first seed was chosen from the one cluster shown to be significant in the AFC > NAC comparison (cluster with a peak in postcentral gyrus). The postcentral gyrus seed was based on peak activation coordinates from within-group analysis in both the AFC and NAC groups, combining two 5 mm spheres around each group’s peak coordinate, thus encompassing surrounding regions that were also activated in these within-group analysis (Fig. 3). Two additional seeds were chosen from the SOR correlational analysis within the AFC group: the thalamus and the lateral prefrontal cortex (Figs. 4 and 5). These particular clusters were chosen for further exploration based on prior research which has implicated both the thalamus and lateral prefrontal in sensory regulation [9, 19, 20]. These seeds were created using the clusters of significant correlations between SOR and functional activation in the two regions of interest. Analyses were thresholded at Z > 2.3 (*p* <.05).

## Results

### Behavioral analysis

Independent sample t-tests showed that youth in the AFC group had significantly elevated SOR anxiety symptoms relative to the NAC group (Table 1). In the AFC group, SOR was significantly correlated with anxiety (*r* =.46, *p* =.01). Seven (22.5%) of parents of the AFC group endorsed 25 or more items on the SCARED, indicating elevated anxiety levels [5, 6]. Based on cutoffs for elevated SOR previously identified by comparing autistic versus non-autistic populations [42], 25% of the AFC group had elevated SOR while no individuals in the NAC group demonstrated significantly elevated SOR scores. Neither SOR nor anxiety were significantly correlated with age of placement in foster care, total time spent in foster care, age of final placement, time in their final adoptive home, or number of placements (r values ranged from − 0.13 to − 0.32 and p values 0.10 to 0.51). More information regarding the sensory sub-domain specific SOR levels in both groups is provided in the supplement (Figure S3).

### Within- and between group neural activation during joint aversive sensory stimulation

**Fig. 1:**
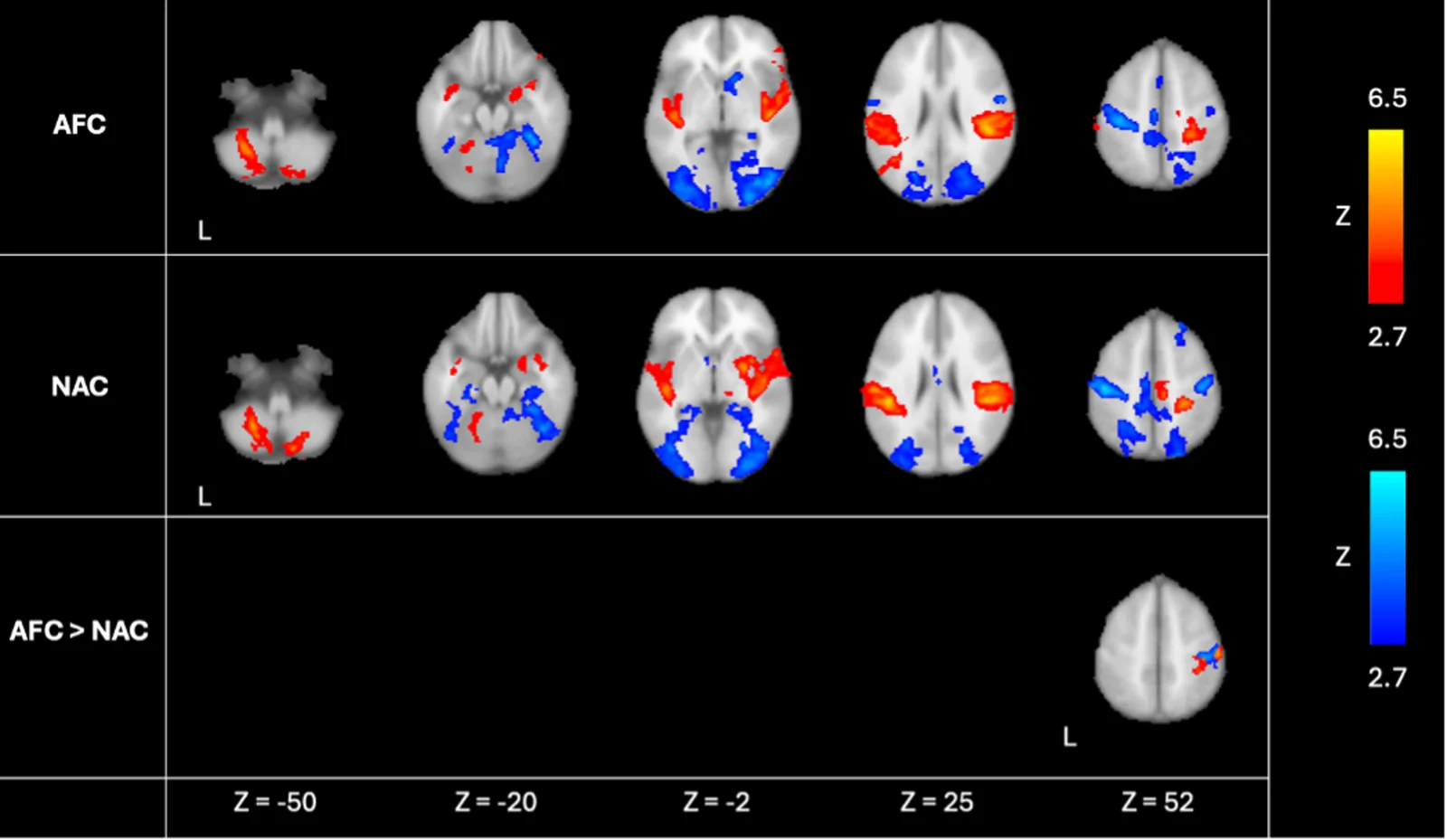
Within- and between-group analysis of whole brain activity during aversive joint sensory stimulation. Sensory-evoked neural activation in children adopted from foster care (AFC) and non-adopted comparisons (NAC). The top two rows display within-group results, with red indicating regions that were more active during sensory stimulation than rest, and blue indicating regions that were less active during sensory stimulation than rest. The third row indicates between-group contrasts. There were no NAC > AFC group results that survived the statistical threshold. The AFC > NAC between-group contrast was masked by within-group results at z > 1.7, with red indicating clusters that were more activated in the AFC group and blue indicating clusters that were more deactivated (i.e., the region is more active during rest than during sensory activation) in the NAC group

Compared to rest, both groups showed increased activation in relevant regions of the somatosensory cortex and bilateral cerebellum during aversive joint tactile and auditory sensory stimulation (Fig. 1; Table 2). Both groups also demonstrated decreased activation of the visual cortices, precuneus and cingulate, compared to rest. The AFC group showed further increased activation of the right inferior frontal gyrus and frontal pole, as well as decreased activation of subcortical structures including caudate, nucleus accumbens, and thalamus. The NAC group showed further decreased activation of frontal regions including the frontal pole as well as middle and superior frontal gyrus during joint stimulation, as compared to rest.

**Table 2:** Cluster peak coordinates

Contrast	Hemisphere	Peak Region	Additional Regions Covered	Voxels	Z-Max	x	y	z
NAC Within Group	Right	Parietal Operculum	Insula, Heschl’s Gyrus	6542	6.77	46	−26	18
	Left	Heschl’s Gyrus	Central Opercular Cortex, Parietal Operculum Cortex	4230	6.34	−50	−18	8
	Right	Postcentral Gyrus	Precentral Gyrus	1279	5.71	30	−38	64
	Left	Crus II	Lobules VII, VIIIa & b	1255	4.95	−24	−84	−42
	Right	Crus II	Lobules VII, VIIIa & b	1017	4.66	22	−88	−42
	Left	Lobules V & VI		431	4.85	−22	−48	−24
	Right	Precentral Gyrus		205	4.97	12	−28	50
AFC within Group	Right	Parietal Operculum	Insula, Heschl’s Gyrus, Frontal operculum	5382	6.77	46	−26	18
	Left	Heschl’s Gyrus	Central Opercular Cortex, Parietal Operculum Cortex	4266	6.34	−50	−18	8
	Right	Postcentral Gyrus	Precentral Gyrus	1856	5.71	30	−38	64
	Left	Crus II	Lobules VII, VIIIa & b	1501	4.95	−24	−84	−42
	Right	Crus II	Lobules VII, VIIIa & b	935	4.66	22	−88	−42
	Left	Lobules V & VI		323	4.39	−30	−44	−28
	Left	Precentral Gyrus		250	4.18	−40	−58	24
AFC > NAC	Right	Postcentral Gyrus		1165	5.26	48	−30	64
AFC: SOR Correlation (negative)	Left	Precentral Gyrus	Postcentral Gyrus, Cingulate Gyrus	3136	4.77	−32	−20	34
	Right	Thalamus (Paraventricular)	Hippocampus	1363	5.36	2	−16	8
	Left	Thalamus (Reticular & Mediodorsal)	Hippocampus, Amygdala	530	4.01	−14	−32	−30
	Left	Lateral Occipital Cortex (superior & inferior)	Angular Gyrus, Middle & Superior Temporal Gyrus	528	3.88	−50	−68	10
	Right	Lateral Occipital Cortex	Occipital Pole	517	3.88	42	−82	−4
	Right	Middle & Inferior Frontal Gyrus	Precentral Gyrus	447	3.57	46	16	30
AFC: PPI Ventromedial Thalamus (negative)	Left	Superior Lateral Occipital Cortex	Precuneus	1406	3.64	−14	−60	58
	Left	Precentral Gyrus		919	3.71	−52	−6	44
AFC: PPI Postcentral Gyrus (positive)	Right	Supramarginal Gyrus	Postcentral Gyrus	420	3.66	24	−26	28
AFC: PPI Postcentral Gyrus (negative)	Left	Intracalcarine Cortex	Lateral Occipital Cortex, Precuneous, Bilateral Medial Cerebellum (Crus II, Vermis, Lobule VII)	4141	3.88	−12	−74	16
	Left	Superior & Middle Temporal Gyrus	Planum Temporale, Supramarginal	2988	4.03	−44	−34	12
	Right	Precentral Gyrus	Precuneus	1292	4.12	8	−44	58
	Right	Posterior Superior Temporal Gyrus	Supramarginal Gyrus	954	3.89	68	−24	0
	Left	Thalamus (Medial Dorsal)		495	3.27	−2	−20	6
	Left	Middle frontal gyrus, precentral gyrus		468	4.04	−48	2	48
	Left	Superior frontal gyrus	Juxtapositional Lobule, Parcingulate gyrus, cingulate gyrus	382	3.25	0	18	58
AFC: PPI Prefrontal Cortex	Right	Occipital Fusiform Gyrus	Lateral Occipital Cortex, Occipital Pole, Occipital Fusiform Gyrus, Lingual gyrus, Bilateral Cerebellum (Crus II, Vermis, Lobule V)	15,983	5.14	32	−62	−12

The Peak Region column indicates cluster peaks, and additional regions covered by cluster are listed in the Additional regions covered column

Voxels indicate cluster size. Z refers to the Z-score at each coordinate. x, y, and x refer to the left-right, anterior-posterior, and inferior superior dimensions, respectively. AFC children adopted from foster care, NAC non-adopted comparisons, SOR sensory over-responsivity, PPI psychophysiological interaction; functional connectivity

The AFC group showed significantly greater activation of sensorimotor cortex (including in the postcentral, precentral, and supramarginal gyri) during joint sensory stimulation compared to the NAC group (Fig. 1; Table 2).

Full statistical details including peak MNI coordinates, cluster sizes and z-statistics are reported in Table 2.

### Correlation between SOR symptoms and neural responses within the AFC group

**Fig. 2:**
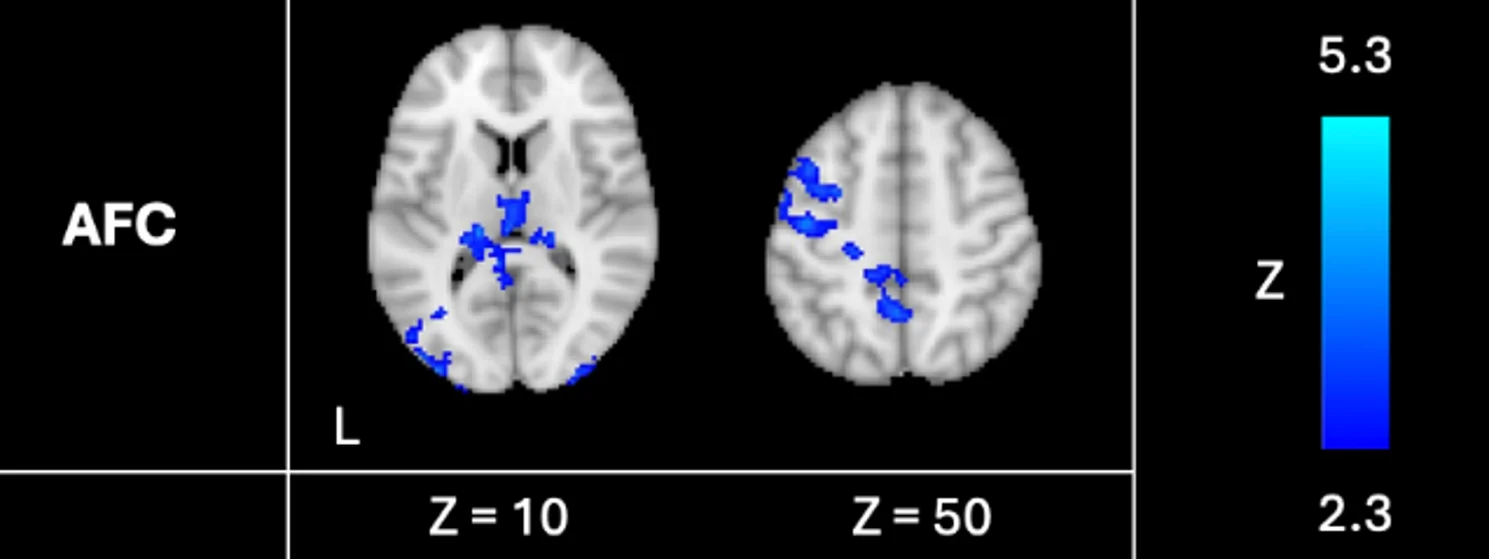
Sensory over-responsivity correlations with sensory-evoked activation in children adopted from foster care (AFC). Blue represents regions where sensory over-responsivity, as indicated by parent reported scores on the Sensory Processing 3-Dimensions inventory, is negatively correlated with sensory-evoked activation

Within the AFC group, SOR symptoms were negatively correlated with greater activation of sensorimotor, frontal and subcortical regions in response to aversive sensory stimulation (Fig. 2; Table 2). Specifically, lower SOR was associated with greater activation in the left precentral gyrus, frontal gyrus, and cingulate gyrus, as well as with greater activation in visual cortex and bilateral thalamus.

### Functional connectivity (PPI)

PPI connectivity analyses were conducted to better understand the functional role of regions identified as being implicated in SOR in the AFC group. Three whole-brain analyses were run. The first analysis was seeded in the postcentral gyrus as this region was hyperactive in the AFC group compared to the NAC group. Two subsequent analyses examined connectivity with the lateral prefrontal cortex and ventromedial thalamus, both regions that were identified in the previous analysis as having increased activation in youth with lower SOR.

**Fig. 3:**
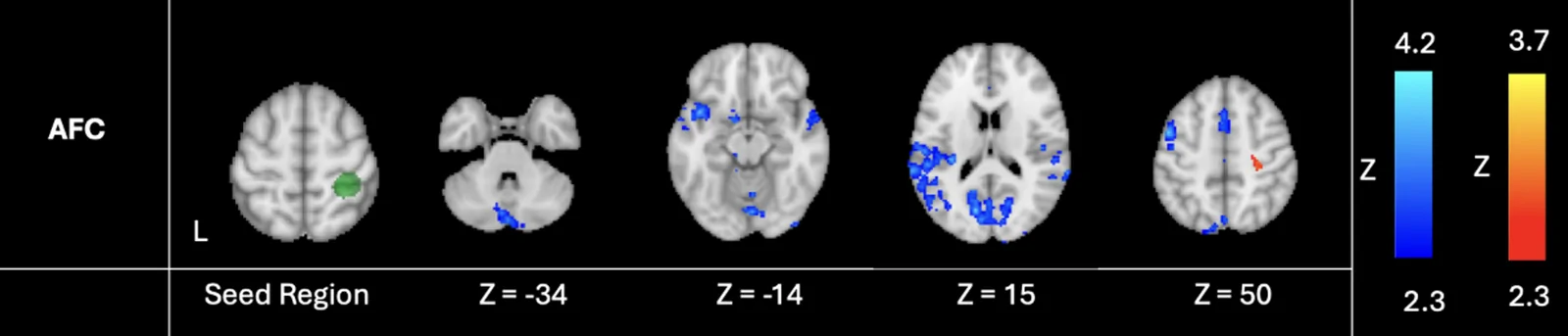
Results from Psychophysiological Interaction Analysis showing functional connectivity of the right postcentral gyrus with the whole brain during sensory stimulation in children adopted from foster care (AFC). Postcentral gyrus seed region (indicated in the leftmost image in green) was created based on peaks of activation identified in the within-group analysis. Blue indicates regions that showed negative connectivity with the postcentral gyrus in response to sensory stimulation, while red indicates regions that showed positive connectivity with the postcentral gyrus during sensory stimulation

*Postcentral Gyrus.* In the AFC group, the postcentral gyrus showed increased negative functional connectivity with multiple sensory-related regions during aversive sensory stimulation compared to during rest. Specifically, during sensory stimulation, postcentral activation was negatively correlated with activity in left pre- and post-central gyri, supplementary motor area, occipital cortex, temporal lobes, and cerebellum. It was also negatively correlated with activity in cognition- and emotion- related regions including precuneus, prefrontal cortex, and paracingulate gyrus, as well as bilateral thalamus (Fig. 3; Table 2).

**Fig. 4:**
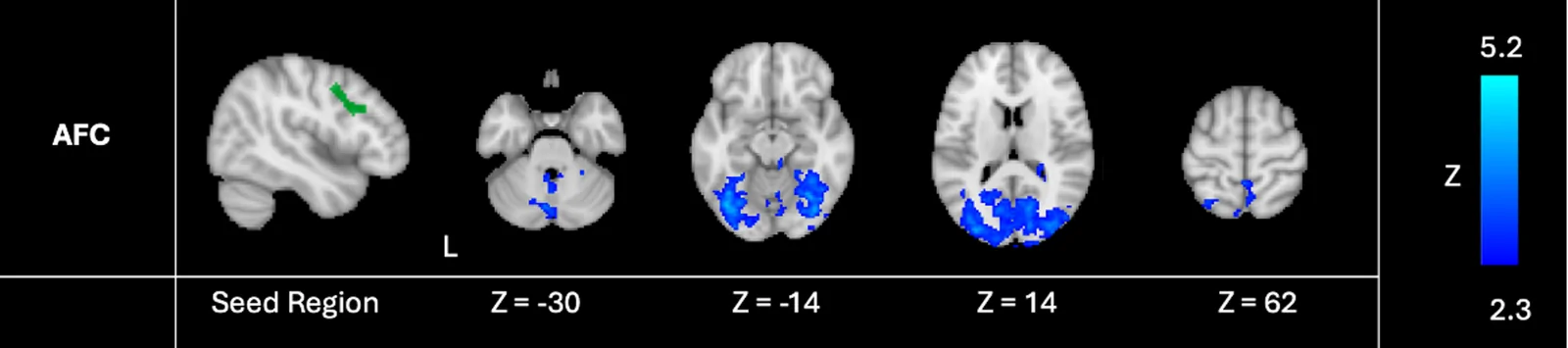
Results from Psychophysiological Interaction Analysis showing functional connectivity of the right lateral prefrontal cortex with the whole brain during sensory stimulation in children adopted from foster care (AFC). Lateral prefrontal cortex (lPFC) seed region (indicated in the leftmost image in green) was created based on voxels where activation was correlated with sensory over-responsivity within the prefrontal cortex. Blue indicates regions that showed negative connectivity with the lPFC in response to sensory stimulation

*lPFC*. During sensory exposure compared to rest, the lateral prefrontal cortex demonstrated negative connectivity with the cerebellum (Crus II, vermis, and lobule V) and large areas of occipital cortex including both primary visual cortex and association regions (Fig. 4; Table 2).

**Fig. 5:**
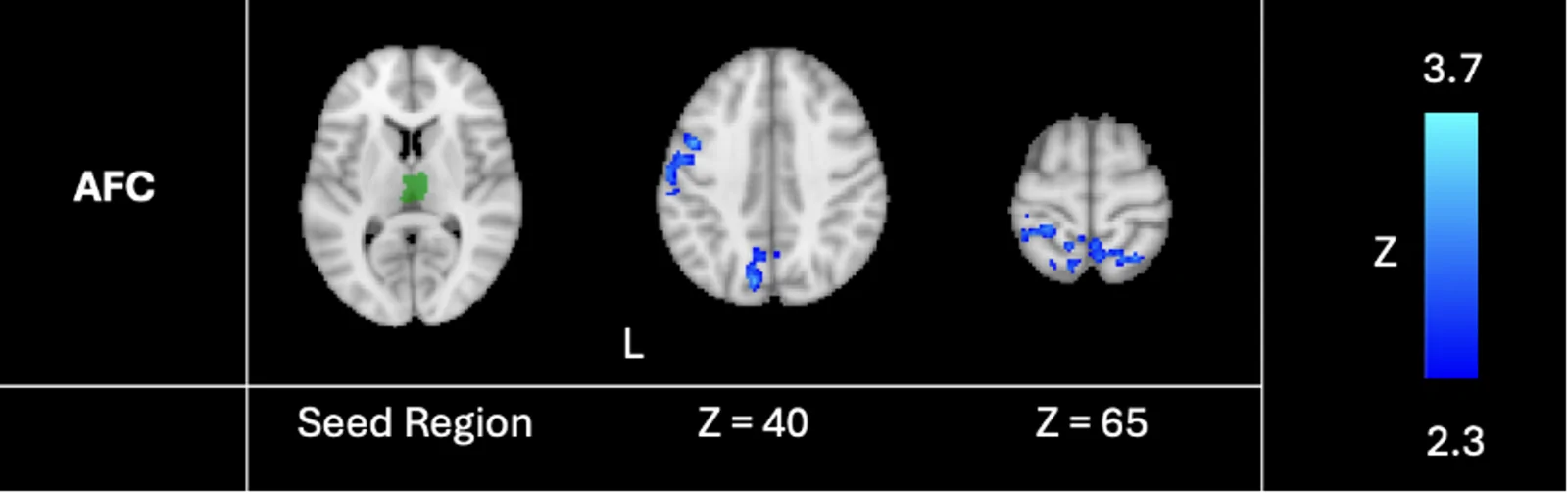
Results from Psychophysiological Interaction Analysis showing functional connectivity of ventromedial thalamus with the whole brain during sensory stimulation in children adopted from foster care (AFC). Thalamus seed region (indicated in the leftmost image in green) was created based on voxels where activation was correlated with sensory over-responsivity within the ventromedial thalamus. Blue indicates regions that showed negative connectivity with the ventromedial thalamus in response to sensory stimulation

*Ventromedial Thalamus.* In the AFC group, in response to aversive sensory stimulation, the ventromedial thalamus showed increased negative connectivity with sensory and motor cortical regions including lateral occipital cortex, pre- and post-central gyri, and superior parietal lobe (Fig. 5; Table 2).

## Discussion

In this study, we examined the relationship between neural responses to aversive sensory stimulation and SOR symptoms in individuals with a history of early caregiving adversity. Results indicated an overall pattern of neural hyperreactivity to sensory stimuli in AFC that is in line with research in other populations with SOR [11, 18, 20]. However, deeper investigation into the neural correlates of SOR and functional connectivity patterns suggests potentially distinct associations between brain responses and sensory reactivity behaviors in this population.

As expected, the AFC group had more severe sensory symptoms compared to the NAC group. This reaffirms previous findings which suggest that children with AFC are at increased risk for sensory-related symptoms [27]. Consistent with these heightened SOR symptoms, the AFC group also showed neural hyperreactivity to aversive auditory and tactile stimulation, specifically in the sensorimotor cortex [16, 27]. To some extent, these findings are in line with SOR studies in other populations and support the idea that SOR may reflect hyperarousal of sensory regions to aversive sensory stimulation across populations [11, 21, 22]. However, in comparison to these other groups, the AFC group overall demonstrated less neural hyperreactivity across the whole brain. The less widespread hyper-reactivity seen in the AFC group could potentially be due to milder (though still elevated) levels of SOR in this group, as well as to additional compensatory mechanisms that reduce the impact of SOR on brain responses for some youth with early caregiver-related adversity.

Subsequent analyses help provide context for such potential compensatory mechanisms. First, we examined how parent-reported SOR severity related to brain responses within the AFC group. We found a negative relationship between SOR scores and neural responses to aversive stimulation, such that AFC youth with lower SOR symptoms had stronger brain activation in prefrontal, sensorimotor, and subcortical regions (e.g., thalamus). The direction of this effect contrasts with previous work in both autism and anxiety [11, 20] which found increased SOR symptoms are associated with greater neural activation. However, the particular regions associated with SOR in this case may play a role in sensory regulation. Specifically, both the thalamus and the prefrontal cortex have been demonstrated in previous studies [19] to play a crucial role in modulating sensory reactivity. Thus, the increased activation in these regions seen in the AFC youth with lower SOR may help explain individual differences in SOR and might point to regulatory mechanisms at play which are mitigating SOR behaviors in some youth with early caregiver-related adversities.

To further explore whether these regions could be playing a regulatory role in the AFC group, we investigated functional connectivity of the postcentral gyrus, ventromedial thalamus, and lateral prefrontal cortex with the whole brain during sensory stimulation. The postcentral gyrus, the one region of significant hyperreactivity in the AFC group, displayed negative connectivity with visual and auditory processing regions in response to aversive sensory stimulation. This finding suggests youth with early caregiver-related adversity may have better discrimination of sensory networks compared to other groups with SOR which typically show hyperactivation across sensory processing regions, even those unrelated to the incoming sensory stimulus [11, 20]. For example, previous studies of SOR in autism have found that in individuals with higher SOR, the visual cortex is hyperactive during tactile and auditory stimulation, suggesting that the visual cortex may lack inhibition in other groups with SOR [20, 28]. Furthermore, in the AFC group, we identified negative functional connectivity between the lateral prefrontal cortex and visual cortex, suggesting that the prefrontal cortex may be downregulating the visual cortex and contributing to the discrimination of sensory networks. This enhanced inhibition of extraneous sensory regions could contribute to resilience against SOR. Consistent with this idea, other studies have found that increased engagement of the prefrontal cortex during aversive sensory stimulation is related to reduced SOR in autism [20, 21] and may underlie age-related improvements in sensory regulation [9]. This is also consistent with other studies that have found that increased engagement of the prefrontal cortex can confer resilience to other internalizing symptoms such as anxiety and depression, which occur at high rates in individuals with early life stress [43, 51]. A similar pattern was evident in connectivity with the thalamus, which is involved in sensory discrimination and mediating cortical arousal [24, 30, 38]. This region showed negative functional connectivity with sensorimotor and visual regions. Previous studies have suggested that reduced thalamo-cortical inhibition may play a role in sensory reactivity in autism [19]. The pattern of negative connectivity observed in the AFC group may suggest that this group is successfully inhibiting cortical arousal of somatosensory regions, again contributing to enhanced resilience to SOR in the AFC group.

This study was the first to examine the neural mechanisms underlying SOR in a group of children with early caregiver-related adversity. However, this study had limitations that should be considered in future research on SOR in populations with early adversity. The population of children adopted from foster care is incredibly diverse, made up of individuals with a vast array of experiences. Although our sample represented many different types of early caregiver-related adversity experiences, it was not large enough to explore how specific types of early adversity may differentially affect sensory symptoms. Additionally, due to the nature of the sample, it was challenging for many adoptive parents to accurately report in detail on adverse experiences that their children may have had prior to placement in their home. Further research on this topic should include a larger sample to investigate the associations between different types and timings of early caregiver-related adversity and later symptoms. Beyond types of early caregiver adversity, this sample contained a great deal of diagnostic heterogeneity, with almost half of the sample taking stimulant medication. As stimulants were observed to influence neural activation in response to sensory stimuli (see Supplement) this study did control for the use of stimulants, but further research is needed to characterize the effect of stimulants on SOR and on the relationship between other psychiatric or neurodevelopmental diagnosis to SOR in this population. Future studies may also aim to investigate the role of sex and SOR outcomes in early caregiver-related adversity groups, as prior research has demonstrated that sex may play a role in sensory processing and symptomatology, as well as in associated symptoms like anxiety. Additionally, it is important to consider both the limitations and advantages of using parent report over self report. Our results replicate levels of sensory behaviors and anxiety seen in prior studies of these symptoms in those with early caregiving-related adversity, and parent report has been shown to accurately reflect many of these observable behaviors; integrating self-report into future studies may provide a more nuanced understanding of the subjective experience of sensory symptoms and other internalizing symptoms in this population [3, 27]. While our study did examine observable behaviors related to sensory processing and anxiety, we did not examine more nuanced day-to-day impairments that may result from SOR, and future research would benefit from a more in depth examination into the functional impairments that result from SOR in this population.

Furthermore, while the AFC group in this study did have higher than average SOR, they demonstrated fewer overall SOR symptoms than previously studied, larger groups with early caregiver-related adversity [27]. This may be due to the self-selecting nature of MRI studies, as individuals with more SOR might be unable to participate due to the uncomfortable scanner environment. However, replicating this study with a larger sample may encapsulate more diversity of sensory symptomology. A larger sample would also allow for investigation of the role of type of and age during adverse experiences on the development and severity of sensory over-responsivity. It is important to note, that in this study we focused on SOR symptoms, however, sensory processing symptoms can manifest in a variety of other ways including sensory seeking, under-responsiveness, and challenges with sensory integration, which may result from early life stress [27].

While studies have examined the neurobiological underpinnings of SOR in clinical groups, to our knowledge, this is the first study to date to examine the neurobiological underpinnings of sensory symptoms in groups with early caregiver-related adversity. Our results demonstrated that children with early caregiver-related adversity do show patterns of neural hyperactivity during aversive sensory stimulation, though in a more constrained region than seen in other groups with SOR symptoms. However, our findings also indicate a unique relationship between SOR and neural activity in youth with early caregiver-related adversity experience that differs from that seen in other groups with SOR. Specifically, we found increased activation in sensory regulation-related regions in youth with *lower* SOR as well as patterns of functional connectivity that suggest these regulatory regions may play a role in increased discrimination of sensory networks. Taken together, these results can help explain individual differences in SOR in youth with early caregiver-related adversity and suggest a potential neural basis for some resilience to more severe manifestations of SOR in youth with early caregiver-related adversity. These results have potential implications for SOR interventions in children with early caregiver-related adversity, which could focus on strengthening the ability to regulate behavior and emotions during aversive sensory stimulation, such as by adapting existing cognitive-behavioral interventions for anxiety [4, 13, 34, 59].

## Supplementary Information

Supplementary Material 1.

## Data Availability

The data that support the findings of this study and the supplementary materials are available from the corresponding author, S.G. upon reasonable request.
