# Supplementary material for: Neural mechanisms of sensory over-responsivity in youth with early caregiver-related adversity
Source: Child Adolesc Psychiatry Ment Health. 2026 Aug 8;20:153. doi: 10.1186/s13034-026-01152-y (PMC13737537; doi:10.1186/s13034-026-01152-y)
Supplement: Supplementary file 1 [file 13034_2026_1152_MOESM1_ESM.docx]

**Supplement**

**Table S1.** Early Caregiver-Related Adverse Experiences in individuals adopted from foster care.

| **Type of Adverse Experience** | **N (Total = 31)*** | **% (of subset)** |
| --- | --- | --- |
| Neglect | 21 | 67.74 |
| Multiple Foster Placements | 21 | 67.74 |
| Prenatal Exposure to Substances | 17 | 54.84 |
| Witnessing Violence in the Home | 12 | 38.71 |
| Physical Abuse | 8 | 25.81 |
| Sexual Abuse | 5 | 16.13 |
| Homelessness | 3 | 9.68 |
| Malnutrition | 1 | 3.23 |
| Failed Finalized Adoption | 1 | 3.23 |
|  | **M (SD)** | **Range** |
| Number of Adverse Experiences | 2.68 (1.25) | 1-6 |
| Time Spent in Foster Care (months) | 13.07(17.66) | 0-71 |
| Number of Placements** | 1.52 (1.63) | 0-7 |
| * 2 participants missing placement and early adversity data, 3 participants missing only caregiver-related adversity data  ** Prior to placement in adoptive home | | |

**Methods**

*Stimulant medication analyses.* In our primary analysis, medication was controlled for in each analysis to ensure that results were not due to medication usage. To better understand how stimulant medication might play a role in sensory processing and sensory over-responsivity, a supplementary analysis was performed to compare neural activation of those taking stimulants with those not taking stimulants within the AFC group. The AFC group used for the original fMRI analysis was divided into two groups, 12 who were taking stimulant medication and 14 who were not. The same methods were used as in the NAC vs. AFC between-group analysis to compare medication vs non-medication groups.

**Results**

*Between-group analysis.* Individuals taking medication showed decreased activation in the right superior temporal gyrus and Heschl’s gyrus during sensory stimuli compared to those not taking stimulant medications (Figure S1).

**Figure S1**. *Between-group analysis comparing sensory evoked neural activation in children adopted from foster care with early caregiver-related adversity (crEA) who are taking stimulants to those who are not taking stimulants.* Red represents regions where individuals who are not taking stimulants have increased activation during sensory stimulation than those who are taking stimulants.

*Correlation Between SOR symptoms and Neural Responses within the AFC grou*p. When anxiety was removed as a covariate from correlational analyses examining the association between SOR symptoms and whole-brain responses to aversive sensory stimulation, the pattern of results remained similar to the analyses with anxiety included as a covariate. SOR symptoms were negatively correlated with greater activation in subcortical regions (bilateral thalamus, putamen, hippocampus, and caudate), as well as sensorimotor and cortical regions (pre- and post-central gyri, cingulate gyrus, supplementary motor area, middle frontal gyrus).

**Figure S2.** *Sensory over-responsivity correlations with sensory-evoked activation in children adopted from foster care (AFC), with anxiety not included as a covariate.* Blue represents regions where sensory over-responsivity, as indicated by parent reported scores on the Sensory Processing 3-Dimensions inventory, is negatively correlated with sensory-evoked activation.

**Figure S3. SOR by Sensory Domain**

**Figure S3.** *Parent Reported Sensory Over-Responsivity Across Domains. ** between group difference p <.05.
